# Postnatal clubs: Implementation of a differentiated and integrated model of care for mothers living with HIV and their HIV-exposed uninfected babies in Cape Town, South Africa

**DOI:** 10.1371/journal.pone.0286906

**Published:** 2023-11-03

**Authors:** Aurelie Nelson, Keitu Lebelo, Tali Cassidy, Laura Trivino Duran, Nompumelelo Mantangana, Leticia Mdani, Nikiwe Malabi, Suhair Solomon, Kate Buchanan, Damian Hacking, Vinayak Bhardwaj, Virginia de Azevedo, Shariefa Patel-Abrahams, Beth Harley, Clare Hofmeyr, Kathrin Schmitz, Landon Myer

**Affiliations:** 1 Médecins Sans Frontières, Cape Town, South Africa; 2 Division of Epidemiology & Biostatistics, School of Public Health, University of Cape Town, Cape Town, South Africa; 3 School of Public Health and Family Medicine, University of Cape Town, Cape Town, South Africa; 4 City of Cape Town Health Department, Cape Town, South Africa; 5 Mothers2mothers, Cape Town, South Africa; 6 J-PAL Africa, University of Cape Town, Cape Town, South Africa; Obafemi Awolowo University, NIGERIA

## Abstract

**Background:**

Despite the overall reduction in the HIV mother-to-child transmission (MTCT) rate in South Africa, poor adherence and retention in care during breastfeeding contribute to this period being a major driver of MTCT in South Africa. To improve this retention, postnatal clubs were created as an integrated, differentiated model of care providing psychosocial support and comprehensive care for the mother-infant pairs (MIP), including HIV and under-5-child services. We describe the implementation of these facility-based clubs and examine its health outcomes in a peri-urban primary health care setting in Cape Town, South Africa.

**Methods:**

In this prospective cohort study, conducted between June 2016 and December 2019, MIPs were recruited into postnatal clubs between 6 weeks and 6 months of age and followed-up until 18 months of age. Outcomes including maternal Viral Load (VL), and children’s HIV testing were compared to a historical control group. Children’s immunizations and maternal sexual and reproductive health outcomes are also described.

**Results:**

During the implementation of the postnatal club study period, 484 MIP were recruited with 84% overall attendance, 95% overall viral load suppression, and 98% overall uptake of HIV infant testing. Compared to historical controls, the club infant rapid test uptake was 1.6 times higher (95% CI: 1.4–1.9) at 9 months and 2.0 times higher at 18 months (95% CI: 1.6–2.6). Through 12 months and between 12–18 months, maternal VL monitoring was higher in the club group compared to the historical control by 1.5 times (95% CI: 1.3–1.6) and 2.6 times (95% CI: 2.1–3.2), respectively, with similar maternal VL suppression. Of 105 infants attending the 12 months visit, 99% were fully vaccinated by one year.

**Conclusion:**

MIP in the postnatal clubs showed better PMTCT outcomes than historical controls with high levels of retention in care. Other outcomes such as immunisation results suggest that integration of services, such as in the postnatal club, is feasible and beneficial for MIPs.

## Introduction

About 90% of women living with HIV globally reside in 22 countries, mostly in Sub-Saharan Africa. Since the implementation of the Global Plan towards the elimination of mother-to-child HIV transmission (MTCT) in 2011, most of these countries have significantly reduced their MTCT rate, with South Africa achieving a 76% decrease in new vertical HIV infections between 2009 and 2014 [1]. Although in the same period, MTCT has drastically decreased in South Africa from 15% to 3.9%, significant challenges to the elimination of MTCT remain [2, 3]. Whilst before the era of ART, a significant proportion of MTCT was intrapartum -and overtime- during breastfeeding, the good uptake and availability of triple antiretroviral therapy for pregnant women has led to a very low intrauterine transmission in South Africa (0.9% nationally in 2016) [4]. Compared with the antenatal period, mothers are far more likely to be lost from care during the postnatal period, resulting in poor adherence, high viral loads. Consequently the majority of new HIV infections occur in children older than six weeks of age. This has resulted in the proportion of new HIV infections attributable to breastfeeding (vs. pregnancy or intrapartum) increasing from 55% to 75% [4–7].

Postnatal retention in care (RIC) of mothers living with HIV is problematic in Southern Africa with disengagement being twice as likely postnatally than antenatally, and resulting in 25–50% drop outs postnatally [8, 9]. Disengagement from care translates into poor adherence, high viral loads and ultimately a higher chance of MTCT [10]. Some of the reasons highlighted for disengagement are health-system related such as: long waiting times at the clinic, high patient volumes, negative experience with health care workers, difficult transition from postnatal care to ART care [11–16]. Other individual- and societal-related reasons are: stigma and non-disclosure of HIV status, travel costs, regimen fatigue, lack of partner involvement, demands of work, and inadequate knowledge of postnatal MTCT [11–16]. Furthermore, the stresses of motherhood make treatment adherence more difficult, due to the added logistical and emotional demands of being a mother as well as the many health visits required for children under five years old, especially if the mother and child’s clinic (and clinic days) are different [17]. These challenges arise in part from a “one size fits all” approach of ART distribution and may be addressed by differentiated models of care accommodating different populations [17].

Added to the poor retention in care of mothers living with HIV postnatally, is the poor retention in care of children in HIV services postnatally, particularly concerning with the latest WHO recommendation to extend breastfeeding to two years of age [17]. In South Africa, although early infant diagnosis testing is effective (93% of infants are tested at birth), the provincially recommended 9 months and 18 months HIV tests have a poor uptake [18, 19], resulting in more than 50% of children living with HIV under two years of age not being diagnosed [20]. Reasons for this disengagement are similar to the ones mentioned for the mother, as well as increased mobility of children between clinics and health districts as well as difficult tracing of the rapid tests results [4, 21]. Of note, a recommended strategy to improve retention in care of MIP is integration of PMTCT services with neonatal, maternal and child health, but its implementation has been variable [3, 22].

In the last five years, facility-based ART adherence clubs have been adopted nationally (and internationally) for stable HIV patients as a differentiated model of care with the dual objective of decongesting health facilities as well as providing holistic care to ART patients [23, 24]. ART clubs have shown good retention in care and virological suppression compared to standard of care [23]. Qualitative studies have shown, amongst other reasons, that peer support was very valuable to the ART club members, leading to a decrease in stigma [25]. By using peer support, these clubs address biopsychosocial needs of patients, not just focusing on a biomedical approach [26]. The WHO recently recommended that “stable” pregnant and breastfeeding women stay or join adult ART adherence clubs [17]. However, for women who are diagnosed with HIV during pregnancy, these guidelines recommend waiting until they are stable on ART before joining ART clubs. In addition, to reach elimination of MTCT, other global guidelines recommend focusing on mothers who interrupted ART and mothers diagnosed late in pregnancy with HIV, amongst other recommendations [7]. A new model of differentiated care, which includes catering for “high-risk” mothers, can meet both global guidelines.

Based on these observations, we created postnatal clubs and implemented them at a primary care clinic in Khayelitsha. This holistic patient-centred model of care is an adaptation of adult ART clubs for all mothers living with HIV and their HIV-exposed infants, with integration of healthcare services as well as focusing on the first 1000 days of the child [27]. In this paper, we describe the implementation of postnatal clubs and examine its MTCT, maternal and child health outcomes.

## Material and methods

### Study design and setting

Based on observations made whilst working in PMTCT at a primary care clinic in Khayelitsha and on previous work on ART clubs for other vulnerable populations, Doctors Without Boarders (MSF) -in partnership with mothers2mothers (m2m) and City of Cape Town (CCT) [28–30]—created, planned, designed and implemented postnatal clubs. We piloted postnatal clubs in a single primary care clinic in Khayelitsha, Cape Town from May 2016 (start of recruitment) to March 2019 (clubs handed over by MSF to m2m and CCT). Khayelitsha, a peri-urban informal settlement close to Cape Town, South Africa, has an estimated population of over 500,000 and a high antenatal HIV prevalence (33%) with a MTCT rate estimated to be <1% at 6–10 weeks; data on subsequent MTCT are not available but the uptake of the 18 months rapid test is approximately 34% [31].

### Description of the implementation of intervention

The postnatal club model is a differentiated and integrated model of care for all mothers living with HIV and their HIV-exposed infants; providing peer-led psychosocial support to this group of women followed by a one stop shop clinical visit addressing the HIV and non-HIV medical needs of the mother and infant. For the infant, this includes offering under-five services like vaccinations, deworming etc.

For the mother, postnatal clubs encouraged integration of sexual and reproductive health services by ensuring all women were offered contraception at every visit.

#### Participants and recruitment

Participants eligible for the postnatal clubs were any mothers living with HIV and their HIV-exposed infants presenting at the clinic when the infants were less than 6 months old. Mothers with active pulmonary tuberculosis (TB), mothers refusing to transfer their care to the same clinic as their infants and children living with HIV were excluded from the postnatal clubs.

HIV-exposed babies between the ages of 6 weeks and 6 months and their mothers were recruited into postnatal clubs. Mother-infant pairs (MIP) were usually recruited at their first postnatal visit at the clinic by the PMTCT nurse and the club facilitator, using a recruitment schedule. Infants were recruited into postnatal clubs organized by birth cohorts; all infants in a club were within 2 weeks of age of each other.

#### Postnatal club operations

Each club contained between 3 to 15 MIP. At each club visit, there was a group counselling session followed by a clinical visit for the MIP. After weighing and TB symptom screening, the facilitator led a group session on HIV-related topics (such as adherence, disclosure) as well as non-HIV related topics (contraception, gender based violence) and on child health as per the first thousand days campaign (e.g. breastfeeding support, mental health education and screening, early childhood development -ECD- education and activities) [27]. Mothers were screened for depression every 6 months with a mental health questionnaire developed by the Perinatal Mental Health Project [32]. After the group session, the MIP saw the club nurse who provided integrated care for the mother and infant. A summary of the components for each session is described in S1 Annex.

Postnatal clubs met monthly in the first 6 months (for close monitoring) then quarterly as per the locally prescribed child health visits from 6 months until 18 months of age. When the child reached 18 months of age, the MIP “graduated” from the postnatal clubs with mothers transitioning as a group into an adult ART club and children going back to standard of care services. Due to the sparse availability of the club room, a complex schedule was designed.

The club nurse wrote ART prescriptions prior to each club, and the club facilitator collected prepacked ART on the day of the club so that mothers could receive their medication at the club and would not have to queue at the pharmacy. During the Christmas holiday, pre-packing increased to 4 months’ supply.

Mothers were asked to attend club sessions that included scheduled 6 monthly Viral Load (VL) testing but were allowed to send someone else (called a buddy) with the baby in her stead on most other visits. If the MIP did not attend a club session, the facilitator was responsible for recalling the mother within 2–3 days and when possible, did a home visit. If the patient did not present to care within 2 weeks of the recall, she was considered as a Did Not Attend (DNA) but could re-join postnatal clubs posteriorly, in an effort to retain the “high risk mothers”. “High risk mothers” (defined as per the local PMTCT guidelines) were encouraged to be part of postnatal clubs but returned monthly to receive extra support as per the guidelines (enhanced counselling support, extra prophylaxis for the children, quarterly VL with referral to the doctor if not suppressing VL) [19].

#### Monitoring and evaluation

Quantitative data were routinely captured from folders and registers into the REDCap electronic data system. Maternal data were captured in a paper register with one register per club. At each postnatal club, the weight of the mother and TB symptoms screen results were entered. The child’s information was recorded in the child’s folder at every visit and captured into the CCT’s electronic medical records (EMR). Both the mother’s and child’s folders had a postnatal club sticker on the front cover and were kept separately from other clinic’s folders as a unit in a crate for ease of retrieval when attending the club. For vaccination status, when children appeared not vaccinated according to the EMR, the child’s folder was retrieved, and the information checked.

Data were captured into RedCap database and analysed using Stata 14 (StataCorp (2015) Statistical Software: Release 14. College Station, Texas StataCorp LP) [33].

#### Human resources and management

The postnatal club model relied mainly on two cadres, the club nurse and the club facilitator. The club nurse was a CCT professional nurse, trained in child and woman’s care as well as trained in the initiation and management of ART. The club nurse’s specific responsibilities included: scripting ART for the mothers, providing integrated clinical care to the MIP (including “high risk” mothers) and overseeing the recruitment and functioning of the postnatal club.

The m2m club facilitator received basic training on PMTCT and facilitation. The club facilitator’s specific responsibilities included: preparing for the club, leading the group sessions (including extra psychosocial support for “high risk” mothers), distributing prepacked ART, completing the club register, administering the mental health screening questionnaire to mothers 6 monthly [34], checking blood results and tracing defaulters.

Other cadres from the clinic involved in the running of the postnatal club included: a pharmacy assistant; a clerk; a doctor and a clinic manager. Club scheduling was also supported by the CCT ART club champion at the district level. No additional funding was required to run the postnatal clubs.

#### Implementation strategy

Before starting the intervention at the clinic, the MSF team reviewed the clinic flow and worked with the clinic team to adapt it to integrate postnatal clubs. To get buy-in from the clinic, the model was presented to the clinic management and to the whole clinic. The clinic management selected a team from the clinic to be trained and to run the club. This team, as well as m2m mentors, did a three-day training on postnatal club and received further trainings on ECD and on mental health. The MSF team was initially involved in running the postnatal clubs and later, in mentoring and coaching the CCT and m2m teams. The model evolved along the way through a participatory process with the clinic team (with monthly meetings, review workshop) to finally be handed over to the clinic team in February 2019 through a handover workshop (with agreed targets).

### Historical controls

For the purpose of comparing the postnatal club MIP outcomes, a group of historical controls was defined as a group of mothers living with HIV and their HIV-uninfected exposed infants who were recruited at birth into another study, from November 2015 to June 2016, at a primary care clinic nearby with similar demographics [18]. The infants were tested at birth for HIV (PCR), the mothers received extra counselling on subsequent infant testing, and the infants were referred back into standard of care. Results of the infants’ subsequent tests and of the maternal viral loads were followed up with no additional intervention other than monitoring the results, and tracing (results of the tracing are not included in this analysis). Infants with a positive HIV birth or 6-week PCR were excluded from this control cohort.

### Analysis

The primary analysis was a comparison of PMTCT results between the club cohort and historical control cohort in the first 18 months of life. Data were accessed for postnatal clubs between 2016 and 2019 and for the historical controls at the time of the study (between 2015–2018). For this particular part of the analysis, we excluded MIP joining the club after 10 weeks of delivery and only included MIP who joined club from November 1^st^, 2015, to December 31^st^, 2018. Children who seroconverted were excluded from subsequent testing denominators. The PMTCT results included: children rapid test uptake and positivity at 9 months (8–10 months old) and 18 months (17–19 months old); maternal viral load monitoring coverage and suppression (VL<400cp/ml) in the first 12 months and between 12 and 18 months. For VL monitoring coverage, club mothers who did not attend the club that day were included in the analysis.

The secondary analysis was a description of PMTCT results postnatal club cohort only. All MIP joining the clubs between June 1^st^, 2016, and March 31^st^, 2019, were included in the analysis. Retention in care analysis was conducted among MIP who joined a club at week 10 (excluding those who joined later), to create a consistent definition of retention at subsequent time points. The denominators at each time point were the number of MIP with enough follow-up time to be due for that visit. Back to clinic (BTC) was defined as mothers going back to standard of care. Did not attend (DNA) was defined as MIP not attending. Loss to follow up (LTFU) was defined as MIP not attending more than two subsequent visits in a row. Transfer out (TFO) was defined as self-report of MIP attending care elsewhere. For maternal VL monitoring coverage, the timepoints used were 6 months (7–180 days after delivery), 12 months (181–365 days after delivery) and 18 months (12–18 months after delivery). The number of “high risk” MIP (based on the 2015 WCP PMTCT definition) was described over 6 monthly timepoints [19]. After the study was completed, a search was done for HIV-positive results for all LTFU infants (looking for positive HIV PCR test or a CD4 count).

For tertiary outcomes, we looked at other services provided as part of the one stop shop postnatal clubs (such as infants’ immunisation, contraception etc). The definition of full immunisation at 12 months of age was based on the national definitions of immunisation [35]. Vaccination coverage was checked on EMR, and if discordant, by auditing folders. Other outcomes such as insertion of intrauterine contraception device (IUCD) was based on the nurses’ own register. Pap smear completion was based on the electronic register. Any patient who had not completed their Pap smear 3 year prior to enrolment into the postnatal clubs was considered eligible as per the national guidelines [36].

### Ethics

Ethical review and approval were provided by the Foundation for Professional Development Research Ethics Committee (FPD REC clearance certificate 5–2017) and University of Cape Town (UCT) Ethics Review Boards (ref 314/2022). The intervention was approved by City of Cape Town (CCT) Research Board (ID number 7899). Because anonymised routine data were used, no written consent was obtained from club participants. The historical controls data collection was conducted in accordance with the approval from Stellenbosch University Ethics committee (HREC N14/06/060), the Western Cape Provincial Health Research Committee and the MSF-Ethics Review Board.

## Results

### Comparison between postnatal clubs and historical controls

As detailed in Table 1, infant testing as well as maternal VL monitoring coverage and suppression was compared between the postnatal clubs cohort and historical controls. As shown in this table, the 9 months rapid test uptake was 1.6 times higher for the infants in the postnatal clubs (95% CI 1.4–1.9) and 2 times higher at 18 months (95% CI 1.6–2.6). Both at 0-12month and at 12–18 months, maternal VL monitoring coverage was higher in the club group compared to the historical cohort by 1.5 times (95% CI 1.3–1.6) and 2.6 times (95% CI 2.1–3.2) respectively. Maternal VL suppression was equivalent at 0–12 months and at 12–18 months between the two groups. One infant seroconverted at one year of age in the postnatal group after becoming LTFU past the 9 months visit when the infant had a negative rapid test; at 6 months his/her mother had a suppressed VL.

**Table 1:** Maternal Viral Load (VL) monitoring coverage and suppression and infant testing in postnatal clubs and historical controls [37].

	Historical controls n = 221	Postnatal clubs n = 141	Risk ratio (95% CI) [Club/controls]
**Infants**			
9 months rapid uptake (8-10mth)	112/221 (51%)	114/141 (81%)	1.6 (1.4–1.9)
18 months rapid uptake (17-19mth)	70/220 (32%)	90/140 (64%)	2.0 (1.6–2.6)
Seroconversions^a^	2	1	
**Mothers**			
0–12 months VL monitoring coverage	149/221 (67%)	140/141 (99%)	1.5 (1.3–1.6)
0–12 months VL suppression	141/149 (95%)	134/140 (96%)	1.0 (0.96–1.1)
12–18 months VL monitoring coverage	65/221 (29%)	107/141 (76%)	2.6 (2.1–3.2)
12–18 months VL suppression	63/65 (97%)	101/107 (94%)	0.97 (0.9–1.0)

^a^ Control group: two infants seroconverted before 18 months. Postnatal club cohort: one infant seroconverted after exiting the club before their 18 months test

In the historical cohort, two infants seroconverted before 18 months of age.

### Looking at the postnatal club cohort only

#### PMTCT results

**Fig 1:**
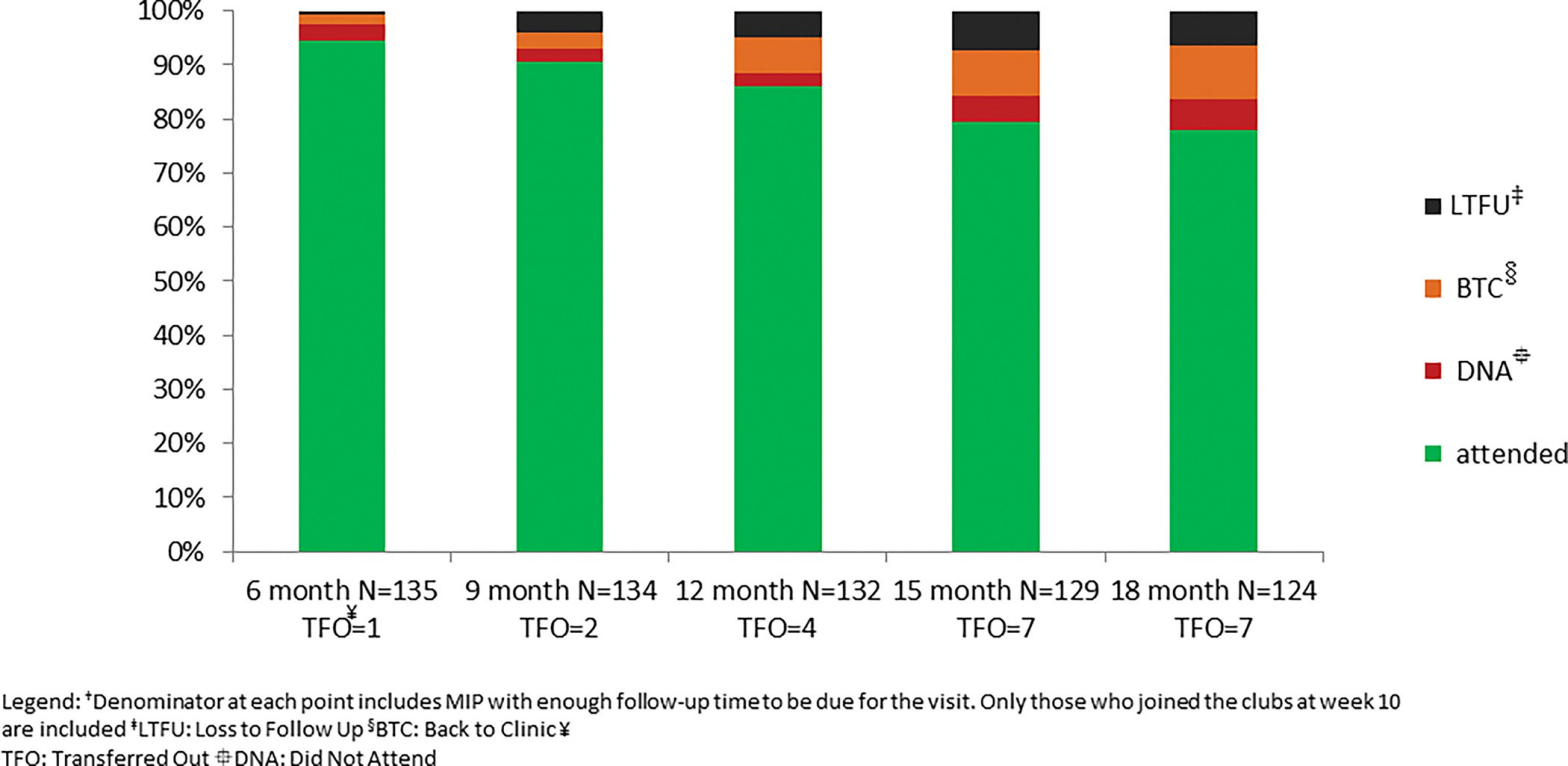
Retention in care of mother and infant pairs (MIP) in postnatal clubs.

During the implementation of postnatal clubs, n = 484 (3 sets of twins) MIP were recruited into the clubs at 10 weeks (baseline). Of those, respectively 353, 238 and 159 MIP had reached and were due for their 6, 12 and 18 months follow up. At 6 months, (318/353) 90% attended. At 12 months, (195/238) 82% attended. At 18 months, (127/156) 81% attended, as shown in Fig 1.

**Fig 2:**
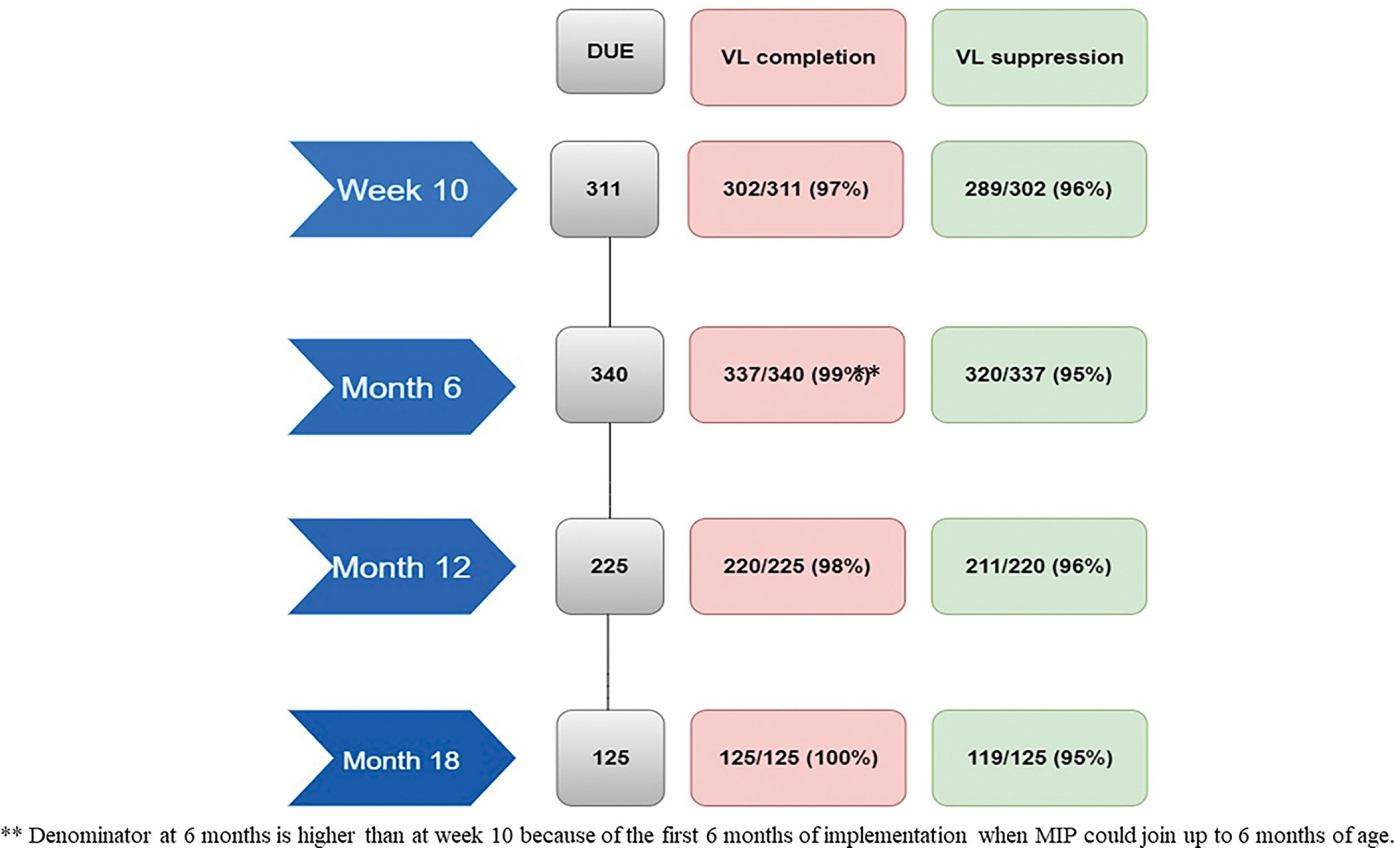
Maternal viral load (VL) monitoring coverage and suppression.

As shown in Fig 2, for the mothers attending their postnatal club visits, maternal viral load monitoring coverage and suppression at baseline (week 10) was respectively 97% and 96%. At month 6, it was 99% and 95%; at month 12, it was 98% and 96% and at month 18, it was 100% and 95%.

As shown in Table 2, of those due and attending the 9 months and 18 months visit, 98% (259/263) had 9 months rapid done and 99% (126/127) had 18 months rapid done. There were no positive HIV results amongst the children attending the postnatal clubs.

**Table 2:** Uptake of infants’ 9 months and 18 months HIV rapid tests (of those attending 9- and 18-months visit) and HIV positivity.

Visit month	Test uptake/ Total attending visit	Uptake percentage	HIV-positive
**Month 9**	259/263	98%	0%
**Month 18**	126/127	99%	0%

As shown in Table 3, the percentage of high-risk MIP was consistent throughout the postnatal clubs: from 6% at baseline to 4–5% at the other timepoints.

**Table 3:** High risk^¥^ mothers at start of the clubs, 6 months, 12 months and 18 months (of those due to attend 10 weeks, 6, 12 and 18 months visits).

Visit month	High risk^¥^ mothers/ Total due to attend visit	High risk percentage
**Baseline**	30/484	6%
**Month 6**	17/340	5%
**Month 12**	9/225	4%
**Month 18**	6/125	5%

^¥^ “High risk” mothers definition is based on the WCP PMTCT definition [19]

#### Vaccination results

Out of a total of 105 infants attending the 12 months visit, 99% were fully vaccinated by one year.

#### Sexual and reproductive health (SRH) and mental health results

Of the 155 mothers who completed the 18 months visit, 125 mothers had Pap smear data available and of those, 31 were not due for Pap smear and of the ones due, (50/94) 53% had their Pap smear during postnatal clubs. Of the 201 women who had an IUCD inserted at the clinic between 2016–2018, 16% (32/201) were part of the clubs. Of those 32, 75% had the IUCD inserted within their first 6 months of the club.

Of the postnatal clubs mothers who were screened for depression, 18% (78/423) overall screened positive at one point for depression on the mental health screening questionnaire.

## Discussion

Looking at a comparison between MIP in the clubs and historical controls, VL monitoring coverage for the mothers between 0–12 months and 12–18 months in postnatal clubs was 99% and 76% compared to 67% and 29%, respectively, for historical controls. High VL monitoring coverage in postnatal clubs suggests that retention in care of mothers living with HIV and their HIV-exposed infants was better in the clubs than for historical controls. VL suppression was comparable between postnatal clubs and historical controls (at 0–12 months 96% and 95% respectively; at 12–18 months: 94% and 97% respectively). Uptake of the 9 months and 18 months rapid tests for the infants in postnatal clubs was 81% and 64% respectively compared to 51% and 32% for historical controls. One child seroconverted (after becoming LFTU) in the postnatal club group compared to 2 children seroconverting in the historical control group.

Analysing the outcomes of the postnatal club cohort only, at 18 months, 81% of the mothers and infants who had started postnatal clubs attended. Of the mothers who attended their 18 months visit, 100% had a VL done and 95% had VL suppressed. 99% of the infants who attended the session had an 18 months rapid test done. All rapid tests were negative. This combined with a 95% full immunization coverage at one year suggests that integration of HIV care and other services is possible for children. Furthermore, although the results for Pap smear and IUCD insertion are difficult to interpret, they also suggest that integration of HIV care and non-HIV care for mothers is possible out.

When looking at retention in care of the MIP, both the comparative results between postnatal clubs and historical controls and the results of the postnatal club cohort suggest a better retention in care of the MIP in the clubs than what would be observed in standard of care. This is in agreement with another study done locally, the MCH-ART randomized controlled trial, which higher RIC of mothers in integrated maternal and child health (MCH) care compared to standard of care (absolute risk difference: 0.21; 95%CI: 0.12–0.30) [38]. Recently, meta-analyses have been looking at RIC of women living with HIV postnatally [39–41]. They have all identified integrated care as a factor improving RIC. The impact of other factors such as peer support, mhealth interventions, and cash transfers are more variable. There are potential multiple reasons for the observed improved retention in care of MIP in postnatal clubs. Duvivier et al’s qualitative study of the postnatal club suggests that mothers enjoyed the strong peer support they received in the club as well as the comprehensive care for themselves and their infants [42]. The mothers felt that the peer support decreased HIV stigma, allowing easier disclosure to family or partners. They also saw the club as timesaving by limiting their trips to the clinic. This is in agreement with a qualitative study done for adult ART adherence clubs as well as studies done in PMTCT on peer support [25, 43]. Britain’s meta-analysis found that peer-led support did not improve RIC but continuity of care with one provider did [40]. Because our study included integration of care as well as continuity of care with one clinical provider and peer support, it is likely that all these factors played a role in improving RIC of the MIP.

Integration of care in the club also led to a very good uptake of vaccination of children and relatively good uptake of reproductive and sexual services. Although the data on IUCD insertion and contraception are limited in this study (with likely some under reporting of IUCD insertion), unmet contraceptive needs is a challenge in South Africa and any attempts to encourage postpartum contraception is welcome to try to space out pregnancies [44]. Other studies in Sub-Saharan Africa looked at integrating postpartum contraception with immunization and also reported it was feasible and welcomed by mothers [45, 46]. Of note, it is part of standard of care for women accessing ART services to be offered contraception, so data were not collected on this. There is also very little literature on the uptake of contraception in ART clubs, except to point out that it is integrated with the services [47].

A major difference from the ART clubs recommended by the WHO for pregnant/breastfeeding women is that postnatal clubs includes “unstable” or “high risk” women. Although “high risk” women in the postnatal clubs received specific interventions (such as monthly visits in between club visits and ART adherence counselling), the results do not show improvement in the number of “high risk” mothers overtime. More research is needed into understanding why the women become “high risk” and how to best support them. Studies in a neighbouring clinic suggested that one third of postpartum women had suffered from intimate partner violence (IPV), associated with a high VL postnatally [48]. Although there were anecdotal reports of IPV by the club mothers, and although gender-based violence was discussed in the group session, the postnatal club intervention did not specifically focus on IPV. As part of the first 1000 days component of the postnatal clubs, mothers were screened regularly for mental health issues, as well as discussing depression in the group session. Although the number of women screening positive on the mental health questionnaire was lower than expected at 18.4%, it is well known that pregnant/breastfeeding women with HIV are at high risk of mental health illness and recommendations have been made globally to address the issue [49, 50]. More in-depth analysis of risk factors for “high risk” women is needed to better tailor interventions to accommodate them. The number of “high risk” mothers being small, it is difficult to draw conclusions from these results. It suggests however that it is possible to have a more flexible type of club, like the postnatal clubs, where “high risk” mothers can be catered for. This will hopefully lead to better RIC of “high risk” mothers and contribute to lowering MTCT in the breastfeeding period.

Looking at the cost-effectiveness of this intervention, we can compare it to the MCH-ART study, which found that integration of maternal and child health in one location was cost-effective and improved the MIP outcomes [51]. Duvivier’s qualitative study suggested that the implementation process of postnatal clubs was feasible to staff, despite initial heavy involvement from the MSF staff [42].

When interpreting these results, there are some strengths and limitations to be considered. Strengths to this study is that it was implementation science, done in a “real life” setting with no reimbursements or financial incentives used, as well as working with the existing team and infrastructure. Furthermore, the intervention was designed and implemented collaboratively with important stakeholders in the health system, such as CCT and m2m. This is likely to have contributed to the good buy-in of the intervention from other clinics and other stakeholders in the health department. Some limitations include the fact that the results presented in this paper are from one peri-urban clinic only and in the Western Cape Province. Although outcomes are generally better in Western Cape Province than other provinces and resources usually more accessible, its HIV prevalence is comparable to the rest of South Africa [53]. After the study ended, the postnatal club model was expanded to two more clinics in Khayelitsha with results on RIC of MIP still pending. Another limitation is that the description of the implementation of the postnatal clubs corresponds to the period when the clubs were implemented by Department of Health staff with MSF staff mentoring. Longer study period without MSF’s support would be advisable to see if the outcomes remain significantly better than standard of care.

Other limitations included using a historical cohort for comparison to the postnatal club MIP. Although using a retrospective cohort brings inherent bias, the PMTCT results from this cohort are very similar to what is reported elsewhere [52, 53]. Furthermore, it is likely that the VL suppression appears similar in both groups because only the most engaged women in the historical group would have gone for VL testing, whilst the least adherent ones would have not, resulting in the likelihood that the proportion of virally suppressed women is lower in the historical group than in the postnatal club group. Confounding factors could include the fact that the MIP recruited into the historical cohort were recruited at birth, whereas the MIP recruited into the postnatal clubs were recruited at their 6 weeks visit, therefore not including the mothers who are less engaged and potentially not returning for the 6 weeks visit. Other limitations in the study included collection methods of the mental health data. Although the postnatal club facilitators received initial mental health training, they did not have further mentoring or coaching and this could have affected their confidence in administering the questionnaire, as suggested by other studies in similar context [54]. This could have resulted in a lower positivity rate on the mental health screening questionnaire than expected, compared to studies in similar settings suggesting 25%-57% of women screening positive for postpartum depression [50, 55].

Looking at the generalisability of postnatal clubs, the model described here has been adapted to different contexts and different components have been added or subtracted. This allowed the model to be piloted by MSF in other Sub-Saharan African settings such as in Mozambique and in eSwatini. In South Africa, the postnatal club model was adopted by the National Department of Health as a “best case scenario” and included in the national PMTCT guidelines [56]. It was then rolled out by PEPFAR, including in Johannesburg and more research is awaited to comment on the results of this roll out [57].

## Conclusion

Integrating mother and child care as well as integrating HIV and non-HIV services into a single clinical care model called postnatal club was highly effective. The postnatal clubs model showed good retention in care of MIP, with good VL monitoring coverage and suppression of the mothers as well as good uptake of the HIV testing of infants. Vaccination uptake for the children was also good. These results as well as the integration of care of the MIP, support of the first 1000 days campaign and peer support have made this intervention desirable to be rolled out at the national level to work towards the elimination of MTCT. Further adaptation of the postnatal club model is possible for use in different contexts and more research is awaited on those outcomes.

## Supporting information

S1 Annex Program for the postnatal club activities and clinical care [58]. (TIF)
