# Supplementary figures and images for: Postnatal clubs: Implementation of a differentiated and integrated model of care for mothers living with HIV and their HIV-exposed uninfected babies in Cape Town, South Africa
Source: PLoS One. 2023 Nov 3;18(11):e0286906. doi: 10.1371/journal.pone.0286906 (PMC10624264; doi:10.1371/journal.pone.0286906)

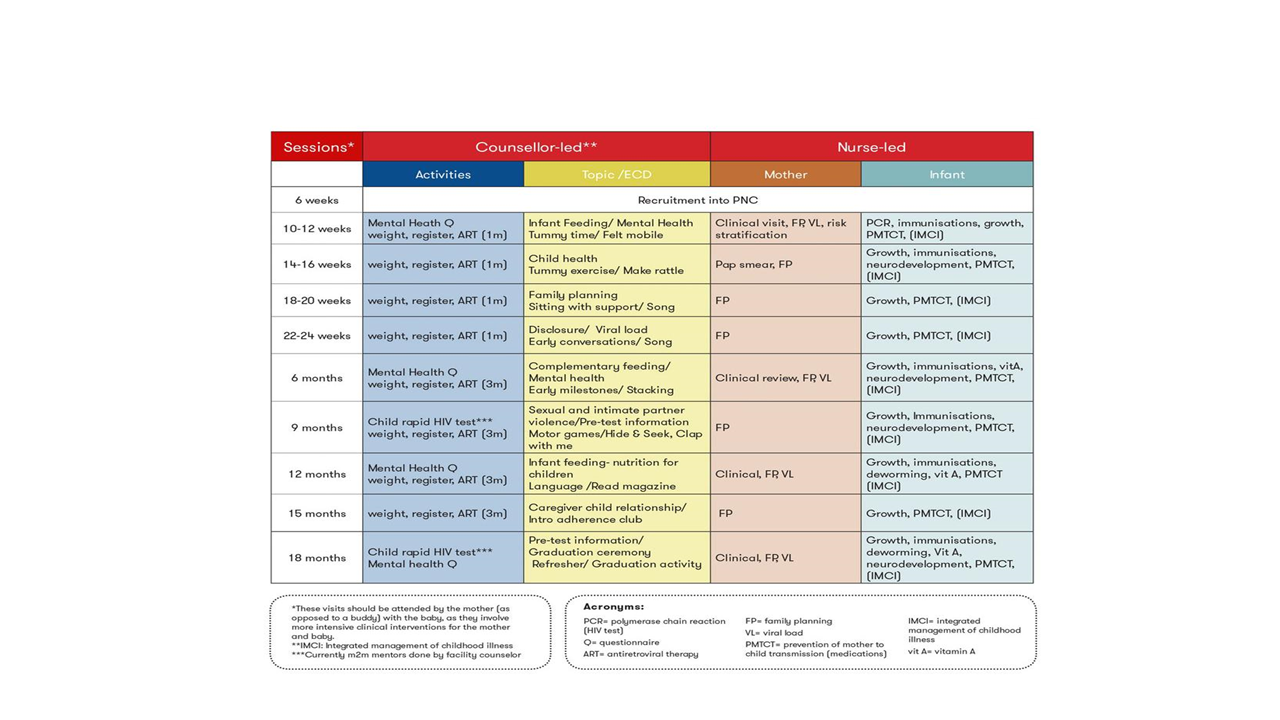

Supplement: S1 Annex — (TIF) [file pone.0286906.s001.tif]
